# Unintentional Discrimination Against Patients with a Migration Background by General Practitioners in Mental Health Management: An Experimental Study

**DOI:** 10.1007/s10488-023-01250-5

**Published:** 2023-01-17

**Authors:** Camille Duveau, Camille Wets, Katrijn Delaruelle, Stéphanie Demoulin, Marie Dauvrin, Brice Lepièce, Melissa Ceuterick, Stéphanie De Maesschalck, Piet Bracke, Vincent Lorant

**Affiliations:** 1 Institute of Health and Society, University Catholic of Louvain, Clos Chapelle-Aux-Champs, B1.31.15, 1200 Brussels, Belgium; 2 Health and Demographic Research, Department of Sociology, Ghent University, Ghent, Belgium; 3 Psychological Sciences Research Institute, UCLouvain, Louvain-La-Neuve, Belgium; 4 Belgian Health Care Knowledge Centre, KCE, Brussels, Belgium; 5 Department of Public Health and Primary Care, Ghent University, Ghent, Belgium

**Keywords:** Migrant, Mental health, Depression, General practitioners, Humanization, Experimental design

## Abstract

Populations with a migration background have a higher prevalence of mental health problems than their native counterparts. They are also more likely to have unmet medical needs and are less frequently referred to mental health services. One potential explanation for this is that physicians, such as general practitioners (GPs), may unintentionally discriminate against migrant patients, particularly when they lack humanization. To date, no experimental study has investigated this hypothesis. This paper assesses the influence of humanization on GPs’ discriminatory decisions regarding migrant patients with depression. A balanced 2 × 2 factorial experiment was carried out with Belgian GPs (N = 797) who received video-vignettes depicting either a native patient or a migrant patient with depression. Half of the respondents were exposed to a text that humanized the patient by providing more details about the patient’s life story. Decisions related to diagnosis, treatment and referral were collected, as well as the time spent on each video and text, and were analysed using ANOVA. Migrant patients’ symptoms were judged to be less severe than those of native patients (F = 7.71, p < 0.05). For almost all treatments, the decision was less favourable for the migrant patient. Humanization had little effect on medical decisions. We observed that GPs spent significantly more time on the vignette with the humanization intervention, especially for the migrant patients. The results indicate that ethnic differences in the management of depression persist in primary care. Humanization, however, does not mitigate those differences in medical decisions.

## Introduction

In Europe, populations with a migration background are at greater risk of mental health problems than native populations, especially depression, post-traumatic stress disorder (PTSD), and anxiety (Ekeberg & Abebe, 2021; Mindlis & Boffetta, 2017; Missinne & Bracke, 2012). Access to appropriate treatment and referral to relevant mental health services have been shown to be limited for migrants (Bhui et al., 2018; Giacco et al., 2014; Kodish et al., 2022).

Migrant populations experience discrimination more frequently, which constitutes an additional mental health risk factor (Levecque et al., 2009; Missinne & Bracke, 2012). Although mental health problems are often identified by primary healthcare services, some studies, however, have shown that general practitioners’ (GPs’) therapeutic decisions regarding patients with a migration background are suboptimal: breakdowns in communication and in the relationship between a migrant patient and a GP can be a frequent source of misunderstanding, the time devoted to a consultation is often shorter for migrants, and GPs’ diagnostic, treatment and referral decisions are sometimes biased by their behaviours and beliefs of migrant patients (Gaya-Sancho et al., 2021; Lepièce et al., 2014; Shannon et al., 2016).

GPs’ beliefs can lead to unintentional discrimination (i.e., unjustifiable differential treatment) against migrant patients with mental health problems. GPs’ decisions are sometimes based on situational context, level of prejudice, cognitive busyness, training, time pressure, implicit/explicit ethnic biases, and their experience of intercultural contact (Gopal et al., 2020; Kite & Whitley, 2016; Lepièce et al., 2014). Poor humanization in care may strengthen these biases. Humanization of care combines the patient-centred care approach and the person-focused care approach (Busch et al., 2019). Humanization includes consideration for the patient’s life story and a holistic approach (more related to the person-focused care approach), as well as empathy, patience, fair-mindedness, equity, active listening, respect for patient’s dignity, uniqueness, and humanity (more related to the patient-centred care approach) (Busch et al., 2019).

To date, humanization in healthcare has received little attention in clinical practice and research. Recently, however, several authors have concluded that more research is needed on the humanization of care by health providers such as GPs and on its impact on their medical decisions (Busch et al., 2019; Pérez-Fuentes et al., 2019). This paper hypothesizes that humanization of care could reduce the risk of unfavourable decisions and reduce unintentional discrimination towards migrant patients with depression. More specifically, by looking at the role of “humanization”, this paper examines a potential explanation for the existence of differences in mental healthcare between patients with a migration background and those without.

To the best of our knowledge, no experimental studies have investigated the effect of humanization on inequalities in the diagnosis and treatment of depression between patients with a migration background and those without. We carried out an experimental study using two staged videos showing a simulation of a GP consulting either a depressed native European patient or a depressed migrant patient. By surveying respondent GPs, we intended to investigate whether GPs treat depressed migrant patients differently to their native counterparts. To do so, we assessed two dimensions of humanization. First, by supplementing videos with life-story text for some GPs, we were able to determine, whether GPs’ decisions reflected attentiveness to their patients’ needs, preferences and life context. Secondly, based on the time respondents devoted to the video and text, we were able to determine, as a proxy of patience, whether, the GPs in the experimental group considered the life story and whether this time differed according to the ethnicity of the patient in the video.

## Methods

### Design

**Fig. 1:**
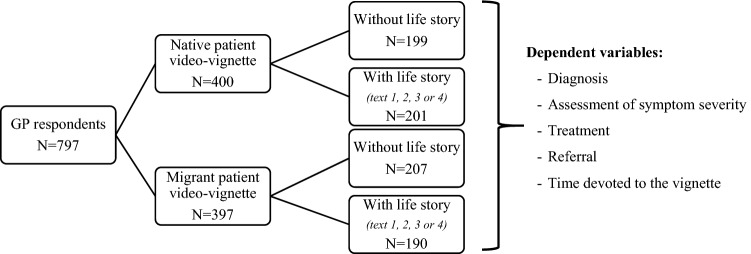
Balanced 2 × 2 factorial experimental design and dependent variables

A balanced 2 × 2 factorial experiment combining migration background and humanization was carried out. Belgian GP respondents were randomly assigned one of the two videos, one of which depicted either a depressed (based on DSM-5 criteria) native patient and one of which depicted a depressed migrant patient. Within those two groups (native patient vs migrant patient), half of the GP respondents received a written text with information about the patient’s life story in order to trigger humanization (see Fig. 1). Hereafter the term “video-vignette” will be used to refer to the video and the text together. The videos and the written introduction are available in Appendix 1. The migration background of the patient was not mentioned, but the actors were typecast for their “Belgian” and “Moroccan” roots, respectively, to elicit implicit ethnic and racial bias and unconscious beliefs.

We created four different life-story texts to be presented to the GPs randomly selected for humanization intervention (see Appendix 2). Each GP in the humanization-intervention, “life-story” group was presented with one of these four texts. The first life-story text introduced the patient by name and included several details about the patient’s family composition, where he lived, and his employment status. The second text provided information about the patient’s needs and treatment preferences. The third text included details of the patient’s job loss. Finally, the fourth consisted of information about the patient’s marital separation. We used four different life-story information in order to avoid the attribution of a changing in medical decisions to one specific information but we wanted to test the effect of life-story information on the whole on medical decisions.

The video-vignettes were similar in every respect, except for the patient’s migration status and the humanization intervention in the written introduction accompanying the video. Within the written introduction, some information about the patient was provided for all vignettes. It was stated that the video simulated a conversation between a GP and a patient who had come for a second consultation due to a persistent headache. Practical details of the methodology are provided in Appendix 3.

### Population

**Fig. 2:**
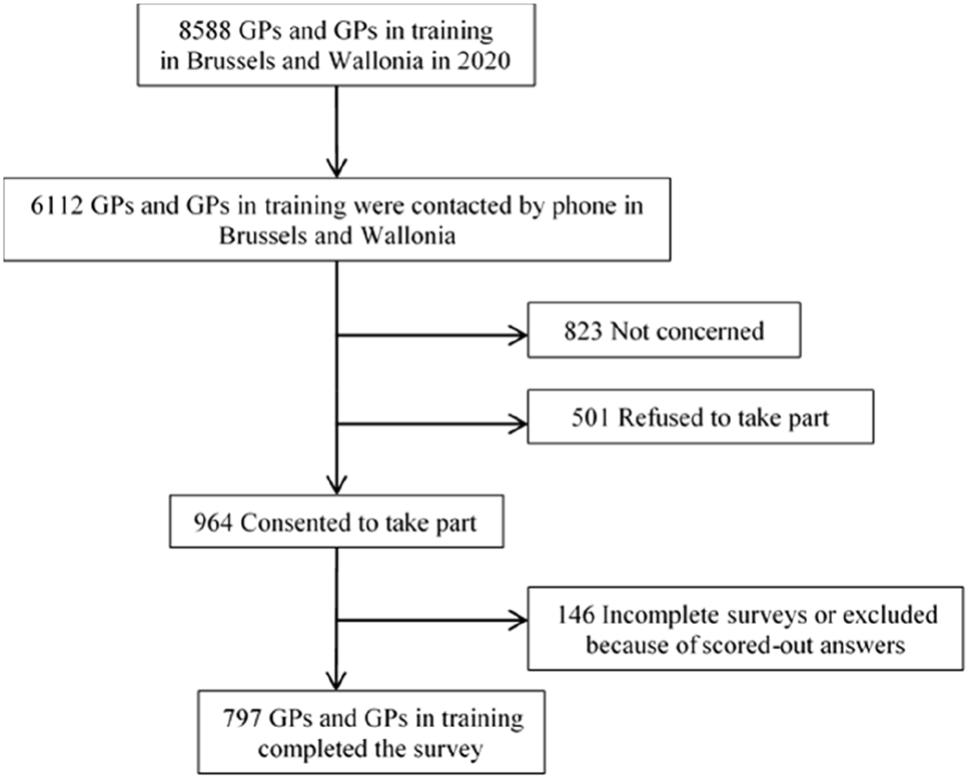
Flow chart showing participation in the sample process

We chose to recruit Belgian GPs. Belgium is an interesting case study because firstly, it is a country with a long history of immigration and secondly, there is a higher prevalence of depression among migrants, especially those from Morocco, and this difference is more significant in Belgium than in other European countries (Levecque et al., 2009; Missinne & Bracke, 2012). The study was carried out with licensed and trainee GPs practising in two of the three Belgian regions: Brussels and Wallonia. Between April and July 2021, we contacted 6112 of the 8588 registered GPs in Wallonia and Brussels by phone. Out of these, we were able to reach 2288 GPs. Among them, 823 GPs were considered as “Not concerned” because they were no longer practising general medicine; we obtained informed consent from 964 GPs, a response rate of 13%. Incomplete questionnaires and questionnaires with scored-out answers were deleted, leaving a final sample of 797 completed questionnaires (Dutch-speaking trainee GPs are included in the final sample). Figure 2 shows a flow chart of the participants in the study.

We also shared the link to the survey in general medicine newsletters and by email with the help of several general medicine associations and other Belgian authorities,^1^ and with Dutch-speaking trainee GPs from Flanders.

### Independent Variables

This paper focuses on two dimensions of humanization: the GP’s consideration for the patient’s life story and the time that the GP devoted to watching and reading the video-vignette, as a proxy of patience. Those two variables constituted our independent variables. We also surveyed GPs’ sociodemographic characteristics, practice, work schedule, and workload, among other factors.

### Dependent Variables

To assess the effect of the humanization and any subsequent difference in GPs’ management of depression in migrant patients, we used four main dependent variables as outcomes: diagnosis, assessment of symptom severity, treatment, and referral.

#### Diagnosis, Assessment of Symptom Severity, Treatment, and Referral

After watching one of the four randomly assigned video-vignettes, respondents filled out an online questionnaire (hosted in Qualtrics®) about potential diagnoses, assessment of symptom severity, treatments, and referrals for their patient. For the diagnosis, respondents had to choose from a list of seven diagnoses of mental health disorders and could tick up to three different diagnoses. We analysed the diagnosis of depression, anxiety, and PTSD. The GPs then scored, on a scale from zero to ten, the severity of the symptoms staged in the video.

For the treatments, we employed a 4-point Likert scale ranging from very unlikely to very likely. First, we asked them what type of treatment they would prescribe, providing three different options: non-medical treatment, medical treatment, or a combination of both. Then, we asked those who had prescribed medical treatment or a combination of both if they would prescribe benzodiazepines (i.e., hypnotics, sedatives, and anxiolytics), because literature shows that GPs prescribe drugs more often to ethnic minority patients (Lepièce et al., 2014). Finally, we surveyed how likely the GP respondents were to refer the patient, also using the 4-point Likert scale.

#### Time Devoted to the Video-Vignette

We measured the time GPs spent reading the introduction and watching the video, using metadata recorded by Qualtrics. The aim of this assessment was to see how the time GPs devoted to the patient differed depending on the ethnicity of the patient in the video and on whether the life-story intervention had been provided or not.

### Data Analyses

#### Characteristics of the Study Population

First, we calculated descriptive statistics. We looked at the sociodemographic distribution of our GP respondents according to sex, age, language, license status, experience, work schedule, workload, type of practice, and the type of area where they practise. Then, we ran several chi-square tests and carried out an analysis of variance using those sociodemographic characteristics to make sure that the allocation of the experimental conditions (migration status and life story) was not affected by selection bias. Table 1 in the results section presents a description of our final sample and the results of the chi-square tests and the analysis of variance.

**Table 1:** Sociodemographic distribution of general practitioner respondents, Belgium 2021 (n = 797) and statistical tests on the allocation of one of the four video-vignettes

Covariates:	% or mean (SD)	Test statistic^a^ (p value)
Sex		0.48* (0.92)
Male	37.0	
Female	63.0	
Age (years)	38.2 (14.8)	0.35** (0.56)
Language		0.22* (0.98)
French-speaking	69.8	
Dutch-speaking	30.2	
License status		4.45* (0.22)
Licensed GP	55.1	
GP in training	44.9	
Experience (years)	12.1 (14.0)	0.11** (0.74)
Working schedule		15.19* (0.09)
Full-time	86.6	
Half of normal working hours	4.5	
< Half of normal working hours	0.9	
Other	8.0	
Workload		9.21* (0.16)
Light	9.9	
Normal	40.1	
High	50.0	
Type of practice (> 50% of their working schedule)		9.93* (0.13)
Unknown	2.5	
Solo	29.5	
Group	66.5	
Other	1.5	
Type of area		1.43* (0.96)
Unknown	0.2	
Urban	11.8	
Sub-urban	27.0	
Rural	61.0	
Vignette		NA
Native patient without life story	25.0	
Migrant patient without life story	26.0	
Native patient with life story	25.2	
Migrant patient with life story	23.8	

*SD* standard deviation, *NA* not applicable

^a^Statistical test was performed on the GPs’ characteristics to check their distribution in the allocation of one of four video-vignettes: “*” indicates the results of a chi-square test and “**” indicates the results of an analysis of variance

#### Diagnosis, Assessment of Symptom Severity, Treatment, and Referral

Secondly, we performed a two-way analysis of variance (ANOVA), which allowed us to gain an understanding of the effects that the two independent variables (migration status and life-story intervention) had on the dependent variables (diagnosis, assessment of symptom severity, treatment, and referral of the patient). We also assessed the interaction effect of the two independent variables on the dependent variables. Then, we computed a multivariate analysis of variance (MANOVA) including all dependent variables at once.

#### Time Devoted to the Video-Vignette

To assess a proxy of GPs’ patience, we analysed whether the time spent on the video-vignettes differed according to the life-story intervention and migration status, using an ANOVA. To do so, we used the difference between the actual average time GPs spent on a video-vignette and the average time expected to be spent on it. This difference was tested with an F-test for each experimental condition. A negative difference meant that GPs spent less time than was, on average, necessary. A positive difference meant the opposite.

All the statistical analyses were performed using SAS 9.4.

## Results

### Characteristics of the Study Population

Table 1 presents the descriptive results of our GP respondents. Women represented 63% of our sample, and the mean age of respondents was 38.2 years (± 14.8). Almost 70% of the GP respondents were French-speakers. More than 86% of the respondents worked full-time and half of the sample perceived their workload to be heavy. Among the respondents 66.5% worked in a group practice, while 29.5% worked mainly in a solo practice. Only 11.8% of our sample worked in an urban area, defined as having more than 75,000 inhabitants; the majority of the GP respondents worked in a rural area (< 10,000 inhabitants). According to the chi-square and the F-tests performed on the sociodemographic variables regarding the random allocation of the vignette, we did not find any selection bias in the randomization of our independent variables (migration status and life-story intervention).

### Diagnosis, Assessment of Symptom Severity, Treatment, and Referral

The results of the two-way ANOVA and the MANOVA for diagnosis, assessment of symptom severity, treatment, and referral are presented in Table 2. No significant differences by migration background or life-story intervention were found for the diagnosis of depression. By contrast, for the native patient, anxiety was more often diagnosed by GPs who had not been exposed to the life story than by those who had been exposed to it (OR 1.63 IC_95%_ [1.10;2.40], result not presented in the table). GPs diagnosed Post-traumatic Stress Disorder (PTSD) more often in the migrant patient and more so in the non-life story group. The assessment of symptom severity was lower for the migrant patient (F = 7.71, *p* < 0.05), with or without the life story.

**Table 2:** Results of the two-way ANOVA and MANOVA for diagnosis, treatment, and referral

	Without life Story		With life Story		Chi-square or F-value (pvalue) Migration background	Chi-square or F-value (pvalue) Life story	Chi-square or F-value (pvalue) Migration background*Life story
	Native	Migrant	Native	Migrant			
	Mean^a^ (SE)	Mean (SE)	Mean (SE)	Mean (SE)			
Diagnosis^b^							
Depression (%)	99 (0.01)	98 (0.01)	98 (0.01)	98 (0.01)	1.06 (0.30)	1.24 (0.27)	0.80 (0.37)
Anxiety (%)	48 (0.03)	37 (0.03)	35 (0.03)	33 (0.03)	2.88 (0.09)	**5.20 (0.02)**	1.59 (0.21)
Post-Traumatic Stress Disorder (%)	9 (0.02)	19 (0.02)	13 (0.02)	14 (0.02)	**4.55 (0.03)**	0.13 (0.72)	**3.97 (0.05)**
Assessment of symptom severity (/10)	7.68 (0.10)	7.45 (0.10)	7.81 (0.10)	7.49 (0.10)	**7.71 (0.01)**	1.16 (0.28)	0.33 (0.57)
Treatment^c^							
Medical treatment	2.97 (0.05)	2.85 (0.05)	2.91 (0.05)	2.77 (0.05)	**4.46 (0.04)**	1.41 (0.23)	0.14 (0.71)
Non-medical treatment	3.67 (0.04)	3.74 (0.04)	3.64 (0.04)	3.71 (0.04)	2.32 (0.13)	0.58 (0.45)	0.01 (0.93)
Combination of both	3.60 (0.04)	3.43 (0.04)	3.57 (0.04)	3.39 (0.05)	**12.2 (< 0.001)**	0.43 (0.51)	0.13 (0.72)
Benzodiazepines (i.e. hypnotics, sedatives, and anxiolytics)	2.06 (0.06)	1.78 (0.06)	1.93 (0.06)	1.77 (0.06)	**8.79 (< 0.001)**	1.37 (0.24)	0.55 (0.46)
Referral							
Likelihood of referral	3.20 (0.05)	3.35 (0.05)	3.32 (0.05)	3.26 (0.05)	0.96 (0.33)	0.12 (0.73)	**4.78 (0.03)**
MANOVA^d^	.	.	.	.	**4.26 (< 0.001)**	0.45 (0.91)	1.46 (0.16)

Results in bold are significant i.e. have a p-value < 0.05

^a^Means, SE (Standard Error), Chi-square, and F-value are adjusted for the GPs’ training

^b^Respondents could choose up to three diagnoses from seven (schizophrenia, bipolar disorder, depression, anxiety, PTSD, obsessive–compulsive disorder, symptomatic and related disorders, and sleep disorders). We have only presented the results for diagnosis of depression, anxiety, and PTSD. Symptom severity was assessed on a scale from zero to 10

^c^Treatment and referral variables were assessed on a 4-point Likert scale from 1: not at all likely to 4: very likely

^d^MANOVA means multivariate analysis of variance

For almost all treatments, the decision was less favourable for the migrant patient than the native patient. The prescription of medication was more likely with the native patient than with the migrant patient (F = 4.46 *p* < 0.05). This result is in line with the observation that GPs were more likely to prescribe benzodiazepines to the native patient than to the migrant patient. This result persisted when controlling for the assessed severity of the symptoms (F = 9.94, *p* < 0.001, result not presented in the table). We did not find significant differences in the prescription of non-medical treatments (F = 2.32, *p* = 0.13).

GPs from the life-story group referred the migrant patient less frequently than GPs from the non-life-story group. We found the opposite for the native patient: there were more referrals from the life-story group than from the non-life-story group (F = 4.78, *p* = 0.03).

Multivariate analysis of variance (MANOVA) indicates that migration status had a significant effect on all dependent variables together (F = 4.26, *p* < 0.01) but that the life story did not (F = 0.45, *p* = 0.91). The interaction effect was not significant (*p* = 0.16)*.* The effect of the life story on the medical decision did not, therefore, differ according to the migration status of the patient.

### Time Devoted to the Video-Vignette

Table 3 shows the results for the difference between the actual time GPs spent on the video-vignette and the time expected to be spent on the video-vignette. Overall, GPs took less time than expected on the four video-vignettes, as the difference was negative. The difference ranged from − 1.76 min (± 0.10) for the native patient video-vignette with life story to − 0.81 min (± 0.08) for the native patient video-vignette without life story. The amount of time GPs devoted to the video-vignette did not differ according to the migration status of the patient (F = 0.35, *p* = 0.56).

**Table 3:** Difference between the actual time (in minutes) devoted to watch the video and read the introduction by GPs and the expected time required to it

	Without life story		With life story		F (pvalue) Migration background	F (pvalue) Life story	F (pvalue) Migration background*Life story
	Native	Migrant	Native	Migrant			
	Mean^a^ (SE)	Mean (SE)	Mean (SE)	Mean (SE)			
Actual timing devoted for each video-vignette (minutes)	3.33 (0.08)	3.58 (0.07)	3.53 (0.10)	3.79 (0.10)	–	–	–
Expected timing for each video-vignette^b^ (minutes)	4.14 (0.00)	4.49 (0.00)	5.30 (0.00)	5.35 (0.00)	–	–	–
Difference between the actual time (in minutes) and the expected time GPs spent on the video-vignette	− 0.81 (0.08)	− 0.91 (0.07)	− 1.76 (0.10)	− 1.56 (0.10)	0.35 (0.56)	**83.43 (< 0.0001)**	3.06 (0.08)

^a^Means, SE (Standard Error) and F-value are adjusted for the GPs’ training

^b^To calculate this average, we added the time of the video to the time needed to read the introduction using a text-to-minutes converter

GPs who had been exposed to the life story spent significantly more time on the video-vignette (F = 83.43, *p* < 0.0001), especially for the migrant patient, where the difference was higher than for the native patient video-vignette, − 1.56 min ± 0.10 and − 1.76 min ± 0.10 respectively.

## Discussion

In our experiment, we found pervasive differences in the diagnosis, assessment of symptom severity, treatment, and referral to mental healthcare services of migrant patients. We therefore conclude that there is a significant association between migration status and medical decisions. These differences are consistent with previous studies that also found that medical decisions were affected by a patient’s ethnic background (Centola et al., 2021; Lepièce et al., 2014). Indeed, providers’ biases have already led to the unsafe undertreatment of migrant patients (Centola et al., 2021). Previous studies have shown that there is a higher prevalence of perceived discrimination among migrant patients, who are also more likely to report communication problems due to their ethnicity (Attanasio & Kozhimannil, 2015; Centola et al., 2021). Patient-provider relationship is one of the main components of patient-centred care and, therefore, of humanization. Regarding our second main finding, unintentional discrimination was rarely reduced by exposure to the patient’s life story, one of the dimensions of humanization. Contrary to our original hypothesis, this result was not consistent with the results of previous studies, which found that more humanized care had a positive influence on several aspects of care such as perceived quality of care, positive health outcomes, and treatment adherence (Swenson et al., 2004).

### Diagnosis, Assessment of Symptom Severity, Treatment, and Referral

Our findings on the higher frequency of PTSD in migrant patient match those observed in earlier epidemiological studies (Aldridge et al., 2018; Ekeberg & Abebe, 2021; Markkula et al., 2017). This could be attributed to the fact that migrants are more exposed to external causes of distress and to disadvantage and discrimination in relation to different social opportunities. When primed with their patient’s life story, however, GPs were less keen to diagnose PTSD in migrant patients. One possible reason for this is that humanization removes the diagnostic shortcut whereby migration is associated with PTSD. Previous studies have found that the main cause of PTSD is great psychological distance from a traumatic event that has become central to a person’s life story (Janssen et al., 2015). Interestingly, we also found in the literature that PTSD risk factors differ considerably between cultures and countries (Bustamante et al., 2017). It is therefore essential that future studies consider the different migration backgrounds and life contexts of patients in relation to PTSD diagnosis.

The lower severity of symptoms attributed to the migrant patient is in line with the findings of a recent study, which are also consistent with those of an experimental study in which physicians concluded that North-African patients were less likely to overreport their symptoms than Western-European patients (Delaruelle et al., 2021; Schulman et al., 1999). Potential explanations for this result may also be related to GPs’ racial prejudice, their dehumanization of migrant patients, a lack of empathy, or even an assumption that Black patients feel less pain than White patients (Trawalter & Hoffman, 2015).

GPs were less likely to prescribe drug treatment or a combination of drug and non-drug treatments to the migrant patient than to the native one but, there was no difference in non-drug treatment. An important finding of this study was that the assessment of symptom severity did not explain the lower likelihood of prescribing drug treatment to the migrant patient because when we controlled the result with this assessment, the result remained unchanged. Previous studies, however, have shown that (unintentional) discrimination was positively associated with severity of mental health problems and negatively associated with therapeutic adherence (Livingston & Boyd, 2010). We think, therefore, that there is a need to make GPs aware that their unconscious underassessment of the severity of migrants’ symptoms may lead to undertreatment, especially for their migrant patients.

GPs prescribed benzodiazepines significantly less often to migrant patients than to native patients. This finding differs from those of a previous epidemiological study on benzodiazepine prescription, which found that migrants from North Africa were more likely to purchase benzodiazepines than the native Finnish-born population (Kieseppä et al., 2022). That study also found, however, that migrants were also more likely to interrupt their treatment before the end of the 6-month period recommended in guidelines on depression (Declercq et al., 2017; Kieseppä et al., 2022). It is possible that GPs presume that the patients will not comply with or purchase their prescription, and therefore decide to prescribe them to migrant patients less often. One explanation for our findings may lie in previous research which shows that GPs consider the prescription of benzodiazepines a form of empathy (Anthierens et al., 2007). The reluctance of GPs to prescribe benzodiazepines to migrants could reflect a lack of empathy towards patients with a migration background.

Contrary to our expectations, GPs were less likely to refer the migrant patient for whom they had been provided with a life story. Qualitative studies might provide some insight. Non-referral by GPs could be explained by a variety of barriers encountered by GPs when attempting to refer their migrant patients. GPs may opt to provide the care themselves because they anticipate that their patients will meet barriers when seeking mental healthcare elsewhere (Teunissen et al., 2015). In the Belgian context, high waiting time or out-of-pocket payment to access a psychiatrist or a psychologist may explain why GPs are reluctant to refer vulnerable patients. In the group without the life story, however, we found that GPs referred the migrant patient more often than the native patient. A possible explanation for this is that GPs feel less comfortable treating severe depression in migrant patients due to a lack of awareness, stigma, or cultural barriers, as highlighted in the literature (Lindert et al., 2008).

### Time Devoted to the Video-Vignette

Our results show that unintentional discrimination in mental healthcare is only slightly moderated by humanization. GPs who had been given the life story, however, spent significantly more time than the controls reading the introduction and watching the video, especially the one with the migrant patient. There are two possible interpretations of this. One is that GPs paid more attention to the context provided by the migrant patient’s life story and humanized him more than the native patient. The other possible interpretation is that GPs who spent more time on the migrant patient’s life story may have felt they were under pressure to diagnose, treat, and refer him in the questionnaire. This possibility is supported by previous research which has shown that restrictions on the time allocated to a consultation can lead to dehumanization (Busch et al., 2019). The negligible effect of humanization on medical decisions may also be explained by the association between dehumanization and GPs’ low perceptions of their own stress or burnout (Capozza et al., 2016). In other words, patient dehumanization can also occur when GPs are under stress due to overwork.

### Strengths and Limitations

This study has several strengths. The first is the innovative and original design of the research. To the best of our knowledge, this is the first experimental study designed to assess the effect of humanization as a moderating factor of GPs’ decisions regarding mental health problems among patients with and without a migration background in a European context.

This study has two important limitations. First, the controlled experimental environment did not replicate the real-life everyday environment of a GP practice. When the use of vignette methodologies for studying health professionals’ decision-making has been assessed, authors have concluded that they are generalizable to “real-life” behaviour (Evans et al., 2015). The controlled design might, however, lead to a social desirability bias in GPs’ responses, particularly in the highly contested domain of migration and health, and thus underestimate the magnitude of actual unintentional discrimination in primary care. Secondly, GPs who received the life-story intervention could have skipped the information that was provided or may not have considered that information in addition to the video, thus jeopardizing the internal validity of the life-story condition. The metadata, however, did not confirm this. GPs spent longer on the patients when the life-story condition was applied. This intervention could be explored using a qualitative design to further explore the effect of this intervention on GP’s everyday practice and to investigate how GPs can use humanization of care to reduce their own ethnic bias.

## Conclusion

In conclusion, we found unintentional ethnic discrimination in assessment of symptom severity, diagnosis, treatment, and referral of mental health problems by general practitioners. As hypothesized in this paper, however, there is no indication that a lack of humanization is the main driver of those discriminatory practices. Nevertheless, we believe that this experiment should be replicated with the humanization intervention staged in different ways. Finally, GPs should be made aware of their under-assessment of the symptom severity of their patients in order to avoid the under-treatment or mistreatment of depression, especially in their patients with a migration background.

## Appendix 1: Video Vignettes in French and in Dutch with the Introductory Text

### Introductory Text

*“After this introduction, you will see a short video. This video simulates a conversation between a general practitioner and a patient, and takes about three minutes to watch. It is the patient’s second consultation due to persistent headaches.*

*Despite an extensive anamnesis, physical examination and a CT-scan, no physical cause for the headaches was found.*

*The patient has no history of psychiatric problems, nor a family history of mental illness. There are no precedents of drug abuse. Besides paracetamol, the patient does not take medication. The patient is currently unemployed.*

*Then, you will be asked a number of questions about a potential follow-up for this patient. Therefore, it is important that you think of the consultation as if it would be taking place in your own practice. The video was developed in collaboration with a medical scientific advisory board consisting of general practitioners, psychiatrists, psychologists and a psychiatric nurse.*

*To watch the video, you will need speakers or a headphone.*

*If the video below does not play correctly, you can watch it via the following link: (one of three video vignettes).*

1. Native Dutch-speaking patient: https://www.youtube.com/watch?v=VLHuXxXNM68.
2. Native French-speaking patient: https://www.youtube.com/watch?v=dvSbtq0GrvU.
3. Belgo-Moroccan Dutch-speaking patient: https://www.youtube.com/watch?v=NA2fKo-2VTs.
4. Belgo-Moroccan French-speaking GPs: https://www.youtube.com/watch?v=6JosPdLTVDs.

## Appendix 2: Texts for Life Story Intervention

- First text: *Mr PEETERS/DUBOIS /ALAOUI,*^2^* grew up with his family made up of a brother and a sister. Currently, he lives in inner-city with his wife and their two children aged one and three years old. He used to work as an office worker.*^3^* He plays football*.
- Second text: *The patient also tells you that he is reluctant to take antidepressants, he is very afraid of side effects*
- Third text: *Six or seven months ago, the patient was under a lot of pressure at work. He and many of his colleagues were laid off from their job as a result of a company restructuring for financial reasons.*
- Fourth text: *You notice in the patient's file that he has been separated for 7 months*.

## Appendix 3: Practical Details About the Methodology

The methodological quality and clinical realism of the vignettes were validated by an academic expert in experimental psychology Delaruelle et al. (2021). For more details on the design, see Ceuterick et al. (2020) and Delaruelle et al. (2021). The script of the vignettes was developed and validated by an academic expert in experimental psychology and an advisory committee composed of two psychiatrists, two GPs, a psychiatric nurse, a psychologist, and an expert in culturally sensitive care. It was stated that the video simulated a conversation between a GP and a patient who had come for a second consultation due to a persistent headache. Physical examination and a CT scan revealed no physical cause for his headaches. The patient had no history of psychiatric problems, no family history of mental illness, and no history of drug abuse and was unemployed. The introductory text and the YouTube links to the video vignettes are available in both French and Dutch in Appendix 1. To avoid any possible bias, the French and Dutch video vignettes were recorded with the same actors, who were almost perfectly bilingual. Some participants in the pilot study started in February 2021, however, questioned the authenticity of the French accent, so we decided to use lip-sync dubbed videos for the data collection in the French-speaking part of the country.

## Footnotes

1 FAMGB (Fédération des Associations des Médecins Généralistes de Bruxelles), Fedasil, SSMG (Société Scientifique de Médecine Générale), FMM (Fédération des Maisons Médicales), ABSyM/BVAs (Association Belge des Syndicats Médicaux/Belgische vereniging van Artsensyndicaten), BHAK (Brusselse Huisartsen Kring), Huis voor Gezondheid Brussel, ICHO (Interuniversitair Samenwerkingsverband HuisartsenOpleiding), HOP (HAIO Overleg Platform), groups for GPs in training on social media, and several medical universities in all three Belgian regions.
